# Pathogenesis of Anorectal Malformations in Retinoic Acid Receptor Knockout Mice Studied by HREM

**DOI:** 10.3390/biomedicines9070742

**Published:** 2021-06-28

**Authors:** Manuel Mark, Marius Teletin, Olivia Wendling, Jean-Luc Vonesch, Betty Féret, Yann Hérault, Norbert B. Ghyselinck

**Affiliations:** 1 CNRS, INSERM, Institut de Génétique et de Biologie Moléculaire et Cellulaire (IGBMC), Université de Strasbourg, 1 rue Laurent Fries, 67404 Illkirch Graffenstaden, France; telma@igbmc.fr (M.T.); olivia@igbmc.fr (O.W.); jlv@igbmc.fr (J.-L.V.); betty2@igbmc.fr (B.F.); herault@igbmc.fr (Y.H.); norbert@igbmc.fr (N.B.G.); 2 Service de Biologie de la Reproduction, Hôpitaux Universitaires de Strasbourg (HUS), 67300 Schiltigheim, France; 3 CNRS, INSERM, CELPHEDIA, PHENOMIN-Institut Clinique de la Souris (ICS), Université de Strasbourg, 1 rue Laurent Fries, 67404 Illkirch Graffenstaden, France

**Keywords:** mouse embryonic development, congenital malformations, vitamin A deficiency, Fraser syndrome, umbilical artery, mesenteric artery

## Abstract

Anorectal malformations (ARMs) are relatively common congenital abnormalities, but their pathogenesis is poorly understood. Previous gene knockout studies indicated that the signalling pathway mediated by the retinoic acid receptors (RAR) is instrumental to the formation of the anorectal canal and of various urogenital structures. Here, we show that simultaneous ablation of the three RARs in the mouse embryo results in a spectrum of malformations of the pelvic organs in which anorectal and urinary bladder ageneses are consistently associated. We found that these ageneses could be accounted for by defects in the processes of growth and migration of the cloaca, the embryonic structure from which the anorectal canal and urinary bladder originate. We further show that these defects are preceded by a failure of the lateral shift of the umbilical arteries and propose vascular abnormalities as a possible cause of ARM. Through the comparisons of these phenotypes with those of other mutant mice and of human patients, we would like to suggest that morphological data may provide a solid base to test molecular as well as clinical hypotheses.

## 1. Introduction

Anorectal malformations (ARM) are a group of diverse congenital defects characterised by an abnormal termination of the rectum, with a clinical spectrum ranging from anal stenosis to persistent cloaca. A majority of the cases are associated with other abnormalities, most frequently urogenital defects. The overall incidence is approximately 1 in 5000 live births [1]. Current understanding of the pathogenesis of ARM remains incomplete. However, analysis of mouse models suggests that the various anatomical presentations of human ARM may depend on the stage of embryonic development at which the dysregulation of cloacal development occurs [2]. The cloaca is a transient dilation of the embryonic hindgut which, in placental mammals, undergoes a complex process of morphogenesis leading to its division into two separate compartments, the urogenital sinus (UGS), at the origin of the urinary bladder and proximal part of the urethra, and the anorectal canal [3,4,5,6].

Diverse environmental factors have been implicated in the aetiology of ARM [1,7] but, so far, little attention has been paid to dietary vitamin A deficiency (VAD). In their pioneering study on the role of vitamin A in embryonic development, Wilson and Warkany (1948) mentioned a delay in the partitioning of the cloaca in vitamin-deficient rat foetuses [8]. A more recent study, also in rats, showed that VAD during pregnancy can result in imperforate anus [9]. All-trans retinoic acid (ATRA) is the active metabolite of vitamin A that plays crucial roles in a large variety of developing processes in the embryo. It is synthesised locally, in its target tissues, through the activity of retinaldehyde dehydrogenases (ALDH1A1, ALDH1A2, ALDH1A3) and acts through binding to retinoic acid receptors (RARA, RARB and RARG; encoded by the *Rara*, *Rarb* and *Rarg* genes), which are ligand-inducible transcription regulators (reviewed in [10]). We have previously shown that mutant mouse foetuses lacking RARA and RARB (*Rara^−^*^/−^*Rarb^−^*^/−^ knockout (KO) mutants) consistently display anorectal agenesis, whereas mutant foetuses lacking RARA and RARG (*Rara^−^*^/−^;*Rarg^−^*^/−^ KO mutants) occasionally display agenesis of the urinary bladder [11,12]. Both the *Rara^−^*^/−^;*Rarb^−^*^/−^ and the *Rara^−^*^/−^;*Rarg*^−/−^ KO mutants additionally show a similar combination of defects in urogenital structures. It was also shown that the cloaca of *Aldh1a2*^−/−^ embryos is severely hypoplastic and malformed [13]. These initial findings suggested to us that the ATRA signalling pathway is important for the development of the cloaca, as well as for that of other structures affected in human ARM.

The present study was undertaken to clarify when the ATRA signalling pathway is required for cloacal development. Because RARs exert redundant functions in a variety of developing systems, we decided to completely impair ATRA signalling through ablation of all three RAR-coding genes. This was not possible by associating *Rara*, *Rarb* and *Rarg* KO alleles in a single mouse, because *Rara^−^*^/−^*Rarg^−^*^/−^;*Rarb*^+/^*^−^* KO embryos do not develop beyond E8.5, precluding any analysis of organogenesis [14]. To circumvent the lethality problem, we recently designed a temporally-controlled, genetic ablation procedure, based on the use of a ubiquitously expressed recombinase (cre/ERT^2^) that can be activated by tamoxifen (TAM) to conditionally invalidate *Rara* and *Rarg* genes in the *Rarb*^−/−^ background [15]. Here, we analysed the phenotypic consequences of ablation of all three *Rar* genes at day 10.5 of embryonic development (E10.5) and at E11.5 on three-dimensional reconstructions from HREM images. Our results indicate that RAR signalling has important functions in the processes of growth and posterior migration of the cloaca. They also provide evidence for a function of RARs in vascular remodelling, and support the possibility that vascular defects during the period of cloacal development could be involved in the pathogenesis of ARM.

## 2. Materials and Methods

### 2.1. Mice

Mice were of a mixed C57BL/6 (50%)/129/SvPass (50%) genetic background. The procedure for conditional ablation of *Rar*-coding genes was described in [15]. In brief, *Rara*^L2/L2^;*Rarg*^L2/L2^;*Rarb*^−/−^ females homozygous for the conditional KO alleles of *Rara (Rara^tm3Ipc^* allele, here noted “L2”; [16]) and *Rarg* (*Rarg^tm3Ipc^* allele, here also noted “L2”; [17]) and for KO alleles of *Rarb* (*Rarb^tm2.1Ipc^* allele, here noted “–“; [18]) were mated with males bearing one copy of the ubiquitously expressed *Tg*(*UBC-cre/ERT2*) transgene [19] and homozygous for L2 alleles of *Rara* and *Rarg* and for KO alleles of *Rarb* (i.e., *Ndor1^Tg^*^(*UBC-cre/ERT2*)/+^;*Rara*^L2/L2^;*Rarg*^L2/L2^;*Rarb*^−/−^). Noon of the day of a vaginal plug was taken as E0.5. To activate the cre/ERT^2^ recombinase in embryos, one TAM treatment was administered to the pregnant females by oral gavage at E9.5 or at E10.5 (130 mg/kg body weight), as described in [15]. This resulted in embryos or foetuses KO for *Rarb*, in which *Rara* and *Rarg* were ablated upon TAM administration when they were bearing *Tg*(*UBC-cre/ERT2*) (hereafter referred to as *Rarabg*^Δ*E10.5*^ and *Rarabg*^Δ*E11.5*^ mutants), as well as their control littermates when the embryos or foetuses were free of *Tg*(*UBC-cre/ERT2*) (i.e., *Ndor1^+/+^*;*Rara*^L2/L2^;*Rarg*^L2/L2^;*Rarb*^−/−^, hereafter referred to as controls).

### 2.2. External Morphology, Histology and HREM Analysis

**Figure 1:**
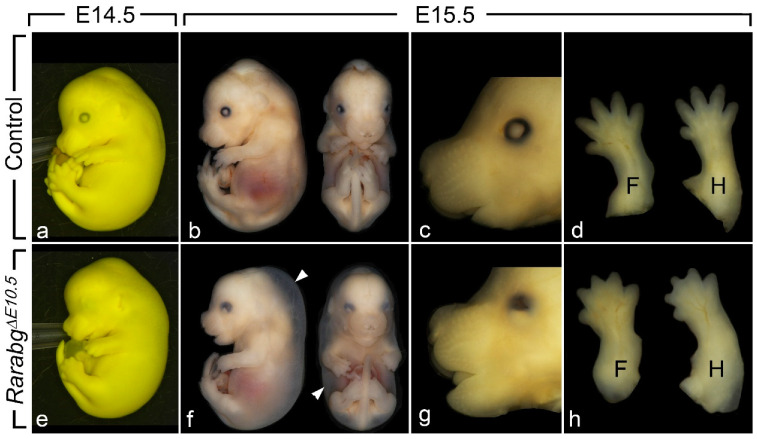
External appearance of control (**a**–**d**) and *Rarabg*^Δ*E10.5*^ mutant (**e**–**h**) littermates at E14.5 and E15.5, as indicated. The cryptophthalmos (**e**,**g**), the generalised oedema (arrowheads in **f**), the shortening of the snout (**g**) and the complete syndactyly (**h**) are characteristic features of the *Rarabg*^Δ*E10.5*^ mutant phenotype. Note that: (i) the two E14.5 littermates (**a**,**e**) are fixed in Bouin’s fluid, which accounts for their yellow and opaque skin, allowing a better visualisation of the cryptophthalmos; (ii) these two E14.5 foetuses were subsequently analysed by HREM (see Figure 2); (iii) the two littermates at E15.5 (**b**–**d** and **f**–**h**) are fixed in paraformaldehyde, which accounts for their translucent skin, allowing visualisation of the subcutaneous oedema (white arrowhead). F, forelimbs; H, hindlimbs.

Embryos treated with TAM at E9.5 were collected at intervals of 24 h from E10.5 to E15.5. Embryos treated with TAM at E10.5 were collected at E14.5 and E15.5. Embryos and foetuses were fixed for 24 h in Bouin’s fluid or in 4% (*w*/*v*) paraformaldehyde, then stored in 70% (*v*/*v*) ethanol. At external inspection, we selected the pairs of mutant and control littermates that were closest in terms of size (e.g., Figure 1a–h). For histology, they were embedded in paraffin, then consecutive, frontal, 5 µm-thick sections were made throughout the entire specimens, and were stained with hematoxylin and eosin. For high-resolution episcopic microscopy (HREM) analysis, embryos, fixed in Bouin’s fluid, were dehydrated and embedded in methacrylate resin (JB-4, Polysciences, Hirschberg an der Bergstrasse, Germany) containing eosin and acridine orange. After polymerisation and hardening, the resin blocks were used for HREM data generation [20,21]. Section thickness was set at 5 (E10.5, E11.5 and E12.5) and 7 μM (E13.5 and E14.5). HREM images were loaded into Fiji [22] to generate virtual stacks. Two-dimensional images were segmented manually with 3D Slicer [23], without interpolation between sections.

## 3. Results

Mouse embryos lacking the three *Rar* genes, either from E10.5 or from E11.5 onwards, were obtained by means of a single TAM administration to pregnant females at E9.5 or E10.5, respectively. This allowed 24 h for complete loss of *Rara* and *Rarg*, that was assessed by immunochemistry and Western blotting [15]. These embryos are referred to as *Rarabg*^Δ*E10.5*^ *and Rarabg*^Δ*E11.5*^ mutants in the rest of the manuscript. They were compared to control littermates, as defined in the Materials and Methods section.

### 3.1. Invalidation of Rars at E10.5 Causes ARM-Related and Other Congenital Defects

*Rarabg*^Δ*E11.5*^ mutants survived until the end of gestation (E18.5) and their phenotypic analysis did not reveal abnormalities apart from mild eye defects and a completely penetrant cutaneous syndactyly (interdigital webbing) affecting all digits of the fore- and hindlimbs [15]. In contrast, *Rarabg*^Δ*E10.5*^ mutants survived at most until E15.5. Upon external examination at E14.5 (n = 5) and E15.5 (n = 3), they all displayed a generalised oedema, complete syndactyly of the fore- and hindlimbs, bilateral cryptophthalmos and a shortening of the snout (Figure 1a–h). The generalised oedema (Figure 1b,f) was consistently associated with myocardial hypoplasia (Table 1), and is a known consequence of cardiac failure which accounts for the death of compound *Rar* KO mutant embryos [24]. The syndactyly was always more patent in mutants analysed at E15.5 than at E14.5, because the normal process of digit separation proceeds rapidly during these 24 h (Figure 1d,h). The cryptophthalmos was manifested by a continuity of the skin over the eyeball, from the forehead to the cheek (Figure 1a,e), and was not an isolated ocular defect, but was instead consistently associated with the set of severe malformations of the eyeball listed in Table 1. The shortening of the snout (Figure 1c,g) reflects the poor development of the nasal cavities (Table 1).

Histological analysis of *Rarabg*^Δ*E10.5*^ mutants at E14.5 (n = 5) revealed many additional congenital defects, never observed in the control foetuses (Table 1). All these defects were previously described in details, in *Rara^−/−^;Rarb^−/−^*, *Rara^−/−^;Rarg^−/−^* and *Rarb^−/−^;Rarg^−/−^* KO mutants [11,12,25,26,27]. Interestingly, the fact that syndactyly, ocular defects and choanal atresia affected all *Rarabg*^Δ*E10.5*^ mutants and were as severe as in *Rara^−/−^;Rarg^−/−^* KO mutants provides a morphological support to the molecular data, proving that the ablations of *Rara* and *Rarg* are complete [15].

**Figure 2:**
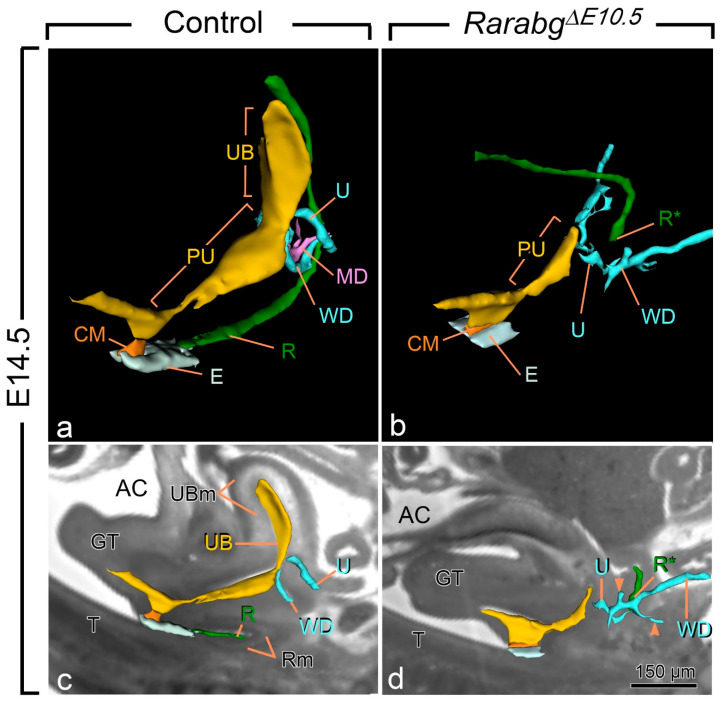
Three-dimensional reconstructions of the rectum and posterior portion of the urogenital tract in a control (**a**,**c**) and a *Rarabg*^Δ*E10.5*^ mutant (**b**,**d**) foetus at E14.5. (**a**,**b**) Ventrolateral views of the left side and (**c**,**d**) projections of left lateral views on sagittal HREM sections. (**a**,**c**) In the control foetus, the urogenital sinus comprises 2 distinct portions: the urinary bladder (UB), which is located anterior to the openings of the ureters (U), and the pelvic urethra (PU) into which the Wolffian ducts (WD) terminate. The pelvic urethra communicates with the amniotic cavity (AC) at the site of disintegration of the cloacal membrane (CM). The rectum (R) is continuous with the tip of the ectodermal groove (E) located between the genital tubercle and the tail. (**b**,**d**) In the mutant foetus, the rectum ends blindly (R*) at a distance from the ectoderm; the urogenital sinus is markedly hypoplastic and lacks the portion corresponding to the urinary bladder. Neither the ureters nor the Wollfian ducts make contact with the urogenital sinus, and the Wolffian ducts give rise to irregular outgrowths (arrowheads in **d**) corresponding to supernumerary ureteric buds; the Müllerian duct (MD) is missing. GT, genital tubercle; R and Rm, lumen and mesenchyme of the rectum, respectively; T, tail; UB and UBm, lumen and mesenchyme of the urinary bladder, respectively. Same magnifications in (**a**,**b**) and in (**c**,**d**). Scale bar in (**d**): 150 µm (**c**,**d**).

All E14.5 *Rarabg*^Δ*E10.5*^ mutants displayed anorectal agenesis (Figure 2a,b; compare R with R*). The urinary bladder (UB) was also consistently missing. Other urogenital structures were absent, malformed and/or or ectopically located: the Müllerian ducts (MD) were missing; the posterior portion of the Wolffian ducts (WD) ended far away from the pelvic urethra and possessed abnormal, supernumerary buds; and the ureters (U) were short and ended blindly at a distance from the UGS (Figure 2a–d). The kidneys were small, malformed, ectopically located and eventually fused together at the midline (Table 1). Kidney defects, as well as abnormalities of the ureters, of the uretero–vesical junction and of the urinary bladder are frequent in human ARM, while Müllerian duct defects occur in 30–45% of girls with ARM [28]. The anorectal agenesis and urogenital defects observed in *Rarabg*^Δ*E10.5*^ mutants were previously shown to occur in all *Rara^−/−^;Rarb^−/−^* and/or *Rara^−/−^;Rarg^−/−^* KO mutants, with the exception of the urinary bladder agenesis, which was observed in only 20% of the *Rara^−/−^;Rarg^−/−^* KO mutants, possibly because of the functional redundancy exerted by the remaining RAR isotype in these mutants [11,12]. The occurrence of urinary bladder agenesis in all *Rarabg*^Δ*E10.5*^ mutants therefore adds morphological evidence to the complete loss of all three *Rar*-coding genes, as previously shown [15].

These results indicate that anorectal agenesis caused by loss of RAR signalling (i) is determined between E10.5 and E11.5, a period corresponding to the onset of cloacal development, and (ii) is always associated with the absence of the urinary bladder, which is another major derivative of the cloaca.

### 3.2. Abnormal Cloacal Development in Rarabg^ΔE10.5^ Mutants

In the mouse, the cloaca appears at E10.5 as a dilation of the hindgut lined by an epithelium (of endodermal origin) and surrounded by mesenchyme (of mesodermal origin), except at the ventral midline where the endoderm contacts directly the surface ectoderm to form the cloacal membrane, that separates the cloaca from the amniotic cavity. Between E11.5 and E13.0, the cloaca divides into a ventral UGS and a dorsal anorectal canal, and it moves posteriorly to reach the midline ectoderm at the base of the genital tubercle. Subsequently, the epithelial duct connecting the UGS and anorectal canal, called the cloacal duct, breaks down, while the cloacal membrane locally disintegrates, exposing simultaneously the anal and urethral openings to the amniotic cavity [3,4,5,6,29].

To clarify the growth pattern of the cloacal region we investigated, on serial histological sections and on three-dimensional (3D) reconstructions from HREM images, the changes in size, shape and position of its derived cavities between E10.5 and E13.5 (n = 2 at each developmental stage). We also segmented other components of the urogenital system frequently altered in human ARMs. We reconstructed the lumen of the gut to assess the possibility that anorectal agenesis may extend anteriorly [30]. The resolution of HREM images did not allow us to visualise accurately the cloacal membrane. Therefore, we segmented the groove between the ectoderm covering the genital tubercle and the tail-bud ectoderm, which provides a landmark for the dorsal limit of the cloacal membrane. As developmental processes in the cloaca and the genital tubercle are interconnected [2,31,32,33,34,35], we interpreted the changes in size and position of the cloaca on sagittal sections through the genital tubercle.

**Figure 3:**
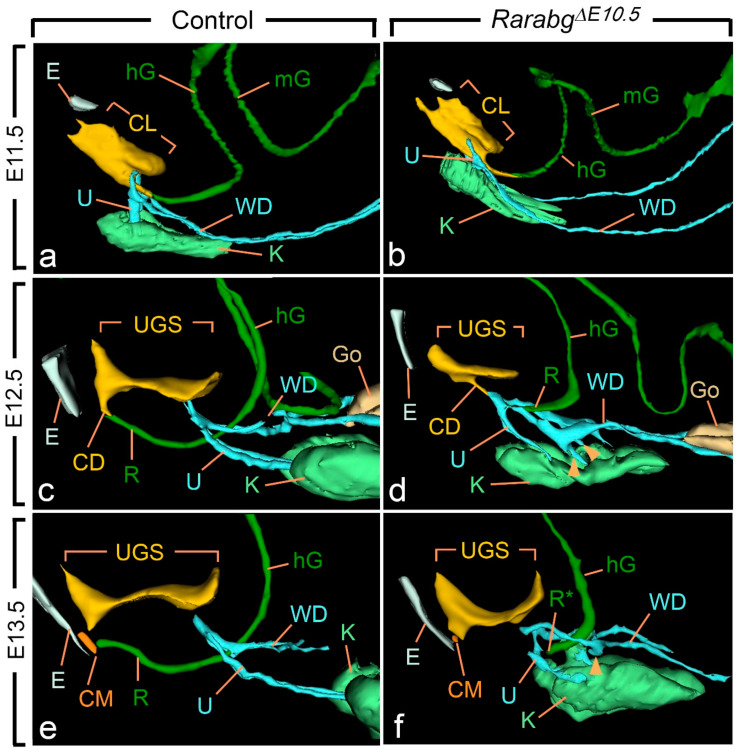
Left lateral views of 3D reconstructions of the urogenital system and gut in control (**a**,**c**,**e**) and *Rarabg*^Δ*E10.5*^ mutant (**b**,**d**,**f**) littermates at E11.5, E12.5 and E13.5, as indicated. CD, cloacal duct; CL, lumen of the cloaca; CM, site of disintegration of the cloacal membrane; E, ectodermal groove between the tail and genital tubercle; hG and mG, lumen of the hindgut and midgut, respectively; Go, gonad; K, metanephric mesenchyme (kidney mesenchyme); R and R*, lumen of the rectum and blind ending of the mutant rectum; U, ureteric buds (**a**,**b**) or ureters (**c**–**f**); UGS, lumen of the urogenital sinus; WD, Wolffian duct. Arrowheads point to abnormal outgrowths of the Wolffian ducts. Same magnifications in (**a**,**b**), in (**c**,**d**) and in (**e**,**f**).

**Figure 4:**
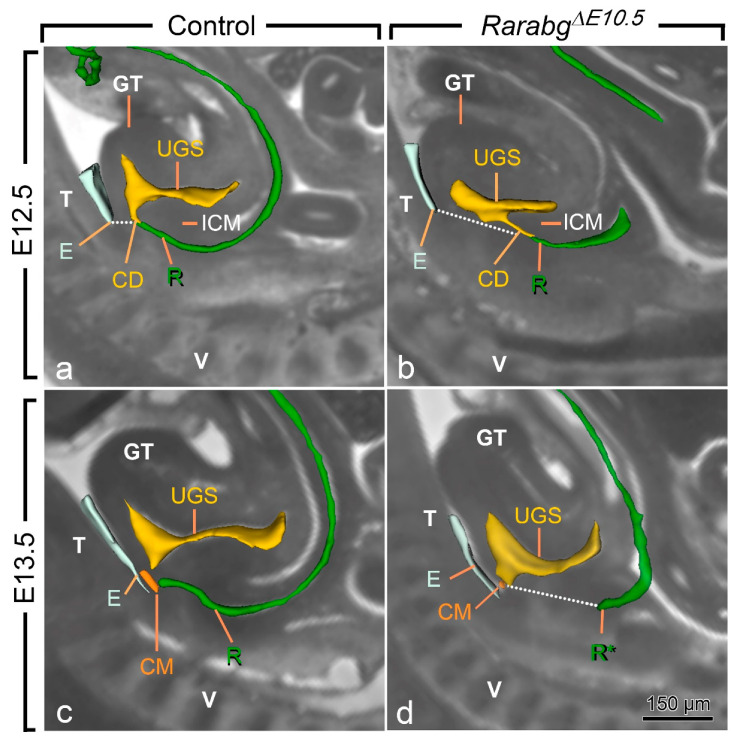
Left lateral views of the urogenital sinus (UGS) and gut in control (**a**,**c**) and *Rarabg*^Δ*E10.5*^ mutant (**b**,**d**) littermates at E12.5 and E13.5 as indicated, with projections on sagittal HREM sections. Note that the layer of mesoderm separating the cloacal duct (CD) and/or the rectum from the base of the genital tubercle is much thicker in mutant embryos than in the controls (dotted lines), whereas the intra-cloacal mesenchyme (ICM), sandwiched between the UGS and rectum, is much less developed in the mutants than in the controls. CM, site of disintegration of the cloacal membrane; E, ectodermal groove between the genital tubercle and the tail; GT, genital tubercle; R and R*, lumen of the rectum and blind ending of the mutant rectum; T, tail; UGS, lumen of the urogenital sinus; V, vertebrae. Scale bar (in **d**): 150 µm (**a**–**d**).

At E10.5 and E11.5, the cloaca of the *Rarabg*^Δ*E10.5*^ mutants and control embryos were indistinguishable (Figure 3a,b and Figure S1). Both had initiated partitioning at E11.5, with the folding of the endoderm at the boundary between the prospective UGS and the anorectal canal (Figure 3a,b). At E12.5, the UGS and the anorectal canal were almost separated, as only a narrow cloacal duct (CD) persisted at the junction between the two structures in both *Rarabg*^Δ*E10.5*^ mutant and control embryos (Figure 3c,d and Figure 4a,b). However, the size of the UGS lumen was reduced and the intra-cloacal mesenchyme sandwiched between the prospective UGS and rectum) was poorly developed in E12.5 *Rarabg*^Δ*E10.5*^ mutants (Figure 3c,d and Figure 4a,b). Moreover, the distance between the urorectal junction (CD) and the tip of the ectodermal groove (E) was markedly increased (dotted line in Figure 4a,b). In E13.5 control embryos, both UGS and rectum (R) had reached the ectoderm (E) at the base of the genital tubercle and they opened separately into the amniotic cavity at the site of disruption of the cloacal membrane (CM) (Figure 3e and Figure 4c). In E13.5 *Rarabg*^Δ*E10.5*^ mutants, only the UGS communicated with the amniotic cavity (at CM), whereas the rectum ended blindly (R*) at a distance from the surface ectoderm (Figure 3f), from which it remained separated by a thick layer of mesenchyme (dotted line in Figure 4d).

Of the other urogenital structures analysed in *Rarabg*^Δ*E10.5*^ mutants at E11.5, the metanephric mesenchyme (K) was normally developed. The Wolffian duct (WD) extended all the way to the cloaca and, before reaching it, gave rise to a single ureteric bud (U) as in the control embryos (Figure 3a,b). At E12.5, however, the mutant metanephric mesenchyme retained its elongated shape and failed to initiate its normal anterior migration, resulting in kidneys that were ectopic (remaining in the pelvis near the bladder), hypoplastic and malformed from this stage onwards (Figure 3c–f; Table 1). The mutant Wolffian ducts displayed small supernumerary ureteric buds extending into the metanephric mesenchyme at E12.5 (Figure 3c,d), which were also observed at E13.5 (Figure 3e,f) and E14.5 (Figure 2a–d). The ureters (U) ended far away from the UGS at E13.5 and E14.5 (Figure 2a,b and Figure 3e,f). The development of the genital tubercle (GT) appeared to proceed normally throughout the period analysed (Figure 2c,d and Figure 4a–d). This was also the case for the abdominal gut segments (hG and mG) located anteriorly to the rectum (Figure 3a–f).

In summary, our developmental analysis shows that the anorectal canal separates from the UGS in *Rarabg*^Δ*E10.5*^ mutants. However, the growth of the UGS is reduced and the anorectal canal does not move towards the ectoderm. This analysis also confirms the consistent association of the anorectal agenesis with defects of the kidneys, Wolffian ducts and ureters.

### 3.3. The Cloacal Defects of Rarabg^ΔE10.5^ Mutants Are Preceded by Abnormalities of Umbilical Arteries

Cell proliferation and apoptosis in both the epithelium and the mesenchyme have been involved in cloacal development [3,4,5,6]. The position of the cloacal epithelium can be reasonably well inferred from surface-rendering views of cavities (Figure 2, Figure 3 and Figure 4). In contrast, the cloacal mesenchyme is not anatomically distinct and thus cannot be readily segmented on HREM images. To integrate mesenchymal structures in our analysis, we took into consideration the position of the umbilical arteries, because their mesenchyme is continuous with the cloacal mesenchyme (Figure S2). To this end, we added the lumens of these vessels and that of their derivatives (the common iliac and posterior mesenteric arteries) in the 3D reconstructions.

**Figure 5:**
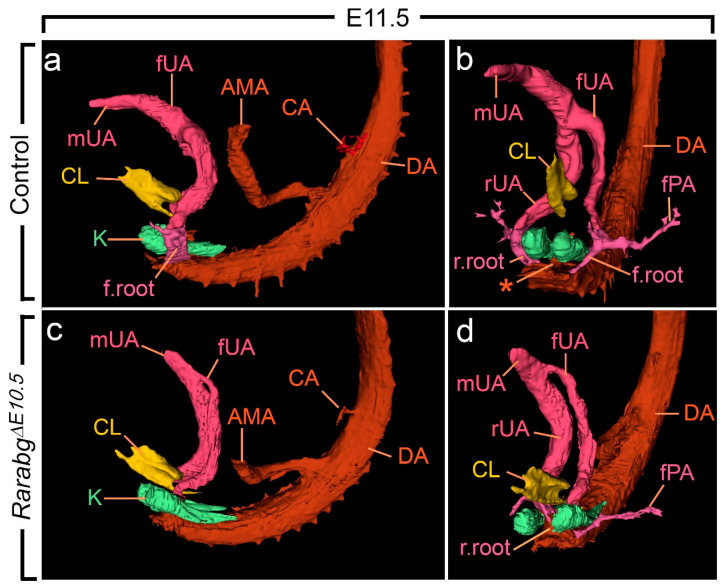
Left lateral (**a**,**c**) and ventral (**b**,**d**) views of the cloaca, kidneys and arteries in control and *Rarabg*^Δ*E10.5*^ mutant embryos at E11.5. (**a**,**b**) In the control, the left and the right umbilical arteries are equal in calibre; their roots have taken a lateral course and bypass the kidneys on each side of the midline; the principal arteries to the hindlimbs arise from their lateral sides. (**c**,**d**) In the mutant, the umbilical roots still originate from the ventral wall of the aorta and pass medial to the kidneys; the left umbilical artery is hypoplastic and the principal artery to the left hindlimb connects directly to the dorsal aorta. AMA, anterior mesenteric artery; CA, coeliac artery; DA, midline dorsal aorta; CL, lumen of the cloaca; K, metanephric mesenchyme; fPA, left artery to the hindlimb bud; r.root and f.root, roots of the right and left umbilical arteries; rUA, fUA and mUA, right, left and middle umbilical arteries, respectively. The asterisk designates the remnant of the medial umbilical roots. Same magnification in (**a**–**d**).

**Figure 6:**
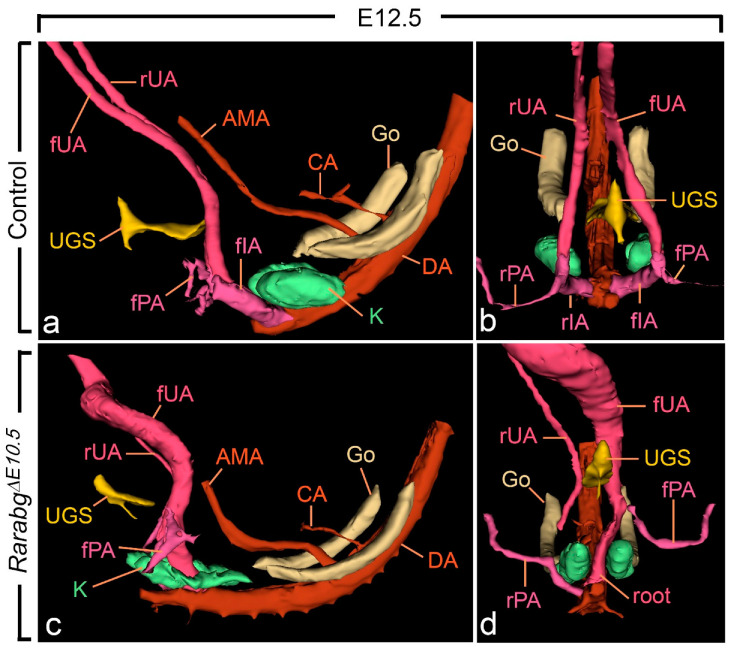
Left lateral (**a**,**c**) and ventral (**b**,**d**) views of the urogenital sinus (UGS), kidneys, gonads and arteries in control and *Rarabg*^Δ*E10.5*^ mutant embryos at E12.5. (**a**,**b**) In the control, the left and the right umbilical arteries have similar calibres; their roots have yielded the common iliac arteries. (**c**,**d**) In the mutant, the right umbilical root has regressed; the left root is in a median position and is hyperplastic as it carries the whole umbilical circulation; the principal artery to the right hindlimb bud connects directly to the dorsal aorta. AMA, anterior mesenteric artery; CA, coeliac artery; DA, midline dorsal aorta; Go, gonad; K, metanephric mesenchyme; rIA and fIA, right and left common iliac arteries; rPA and fPA, right and left arteries to the hindlimb buds; root, root of the single umbilical artery in the mutant; rUA and fUA, right and left umbilical arteries. UGS, lumen of the urogenital sinus. Same magnification in (**a**–**d**).

From E10.5 to E11.5, the paired roots of the umbilical arteries underwent a sudden change of course [36,37]. At E10.5, these roots originated from the ventral wall of the aorta, then they passed medial to the Wolffian ducts, diverged round the cloaca, came together and united ventrally to form a single vessel (Figure S2a,b). At E11.5, the roots arose from the lateral walls of the aorta and then they passed dorsal to the metanephric mesenchyme (Figure 5a,b; Video S1). At this stage, the medial roots had regressed, but their proximal origin was still identifiable (asterisks in Figure 5b). At E12.5 and E13.5, the lateral roots, now termed the common iliac arteries, supplied the placenta, via the left and right umbilical arteries, and the hindlimbs, via their left and right principal arteries (Figure 6a,b and Figure S3a,b; Videos S2 and S3); concomitantly, the medial roots had remodelled into the posterior mesenteric artery (Figure S3a,b).

As anticipated, at E10.5, *Rarabg*^Δ*E10.5*^ embryos were morphologically undistinguishable from control embryos (Figures S1a,b and S2a–d). However, in *Rarabg*^Δ*E10.5*^ embryos at E11.5, the roots of the umbilical arteries had not shifted to a lateral position, resulting in the absence of the common iliac arteries and of the posterior mesenteric artery at later developmental stages (Figure 5c,d and Figure 6c,d; Video S4). In addition, in *Rarabg*^Δ*E10.5*^ embryos analysed at E12.5, one root had regressed completely (Figure 6c,d). Accordingly, a single median vessel connected the dorsal aorta to the placenta from this stage onwards (Figure 6c,d and Figure S3c,d; Videos S5 and S6). On the side where the umbilical root regressed, the hindlimb artery had established a direct connection with the dorsal aorta (Figure 6d and Figure S3c,d).

Altogether, these data reveal a hitherto ignored role of RAR signalling in the development of arteries destined to the placenta, the pelvic organs and the hindlimbs.

## 4. Discussion

### 4.1. A Critical Window of Time for RAR Signalling in Organogenesis

That signalling through RAR regulate complex gene networks involved in growth, morphogenesis and cellular differentiation was inferred from the phenotypic analysis of mice carrying null alleles of two RAR isotypes from the one cell stage embryo onwards. These *Rara*^−/−^;*Rarb*^−/−^, *Rara*^−/−^;*Rarg*^−/−^ and *Rarb*^−/−^;*Rarg*^−/−^ KO mutants display a large array of congenital malformations affecting the cardiovascular, respiratory and urogenital systems, the brain, sense organs, ventral body wall and craniofacial structures (reviewed in [10]). The generation and phenotypic analysis of compound *Rar* KO mice has provided valuable insights on the functions of these receptors during development, but it has intrinsic limitations, mainly due to the introduction of mutations in the germ line. In fact, the earlier the mutation, the more the development of a precursor tissue is impaired, which then prevents the interpretation at the level of individual organs. Moreover, the effect of a germ-line mutation may be functionally compensated for during development, thus precluding the appearance of a defect. To overcome these limitations, we have designed a strategy for temporally controlled somatic mutagenesis of *Rar*-coding genes [15].

The present analysis indicates that the RAR signalling pathway is required for many developmental processes that are determined after E10.5, but before E11.5. Such processes are revealed by the congenital abnormalities of *Rarabg*^Δ*E10.5*^ mutants which (i) equal or exceed, in frequency and eventually in severity, those previously reported in *Rar* KO mutants; and (ii) are not observed in *Rarabg*^Δ*E11.5*^ mutants. We thus demonstrate that E10.5 to E11.5 spans a critical developmental period during which the RAR signalling pathway is required for the formation of the ocular and nasal structures, salivary glands, Müllerian ducts, posterior portion of Wolffian ducts and urinary bladder, as well as for the ascent of the kidney and for the regulation of cloacal morphogenesis (Table 1). Our data also indicate that RAR signalling is required after E11.5 for terminal steps in ocular morphogenesis [15,38] as well as for the separation of the digits [26].

Some defects of *Rarabg*^Δ*E10.5*^ mutants appear less frequent than in the *Rar* KO mutants; this is the case for lung hypoplasia and for persistent truncus arteriosus (Table 1). One possible explanation for the milder occurrence in *Rarabg*^Δ*E10.5*^ mutants is that these defects are determined earlier than E10.5. In agreement with this possibility, the development of the respiratory system can be inhibited by pharmacologically blocking the RAR signalling pathway at the onset of appearance of the primary lung bud outgrowths from the foregut (i.e., at E9.5), but not at later developmental stages [39]. Along the same lines, studies of VAD rat embryos indicate that normal aorticopulmonary septation can be restored if vitamin A is provided maternally at the developmental equivalent of mouse E9.5–E10.5, but not at later stages [40].

Lastly, several congenital defects present in *Rara*^−/−^;*Rarb*^−/−^ and *Rara*^−/−^;*Rarg*^−/−^ KO mutants are not recapitulated upon invalidation of all three *Rars* at E10.5 [10]. Once again, the most likely explanation is that RAR are required earlier in development. Along these lines, a preliminary analysis indicated that embryos in which all three *Rar*ware invalidated from E9.5 onwards (i.e., *Rarabg*^Δ*E9.5*^ mutants) displayed severe craniofacial defects (Figure S4a–d), similar to those observed in *Rara*^−/−^;*Rarg*^−/−^ KO mutants [14,27]. These *Rarabg*^Δ*E9.5*^ mutants also present a very severe myocardial hypoplasia and the majority of them die within 24 h, which makes their analysis particularly challenging.

### 4.2. ATRA-Dependent Events in Cloaca Development

In the absence of all *Rars,* the partition of the cloaca proceeds to completely separate the UGS and the anorectal canal in due time. However, the posterior moving of the dorsal cloaca seems to be delayed or arrested, thus precluding the connection of the rectum to the surface ectoderm. Moreover, the epithelial and mesenchymal portions of the UGS are hypoplastic and the urinary bladder does not form. These results strongly suggest that the RAR signalling pathway is required for the growth and posterior moving of the cloaca, but is not involved in the partitioning process per se. This is in contrast to other signalling pathways. Actually, mice with disrupted sonic hedgehog (SHH) signalling or carrying loss-of-function mutations of *Wif1*, *Dkk1*, *Ephrin2* or *Bmp7* usually display a persistent embryonic cloaca in the form of a communication between the UGS and the rectum [2,31,33,34,35,41]. Interestingly, *Wnt5a* mutants can display a blind-ended rectal pouch without a fistula [32], similar to that displayed by our *Rar* mutants. Our observations further indicate that the gut defects do not extend to more proximal segments of the colon when RAR signalling is invalidated.

Similarly to *Rarabg*^Δ*E10.5*^ mutants, *Hoxa13*-null mice exhibit hypoplasia of the cloaca, abnormal ureteral openings and agenesis of urinary bladder and Müllerian ducts, as well as premature stenosis of one umbilical artery [42]. Interestingly, *Aldh1a2* expression is regulated by HOXA13 in vivo [43]. Taken together, these data suggest that the ATRA-activated RARs can exert functions in cloacal development downstream of *Hoxa13*. Reciprocally, *Hoxa13* expression can be controlled by RAR signalling. Actually, using data sets locating RAR-occupied sites genome-wide in several mouse cell types [44,45], we noticed three robust RAR-binding sites near the *Hoxa13* locus (Figure S5). Along these lines, we also noticed RAR-binding sites in the first intron of *Wnt5a* (Figure S5). Interestingly, the DNA fragment located in *Wnt5a* contained a consensus retinoic acid response element (i.e., direct repeat of two core motifs 5’-RGKTCA-3’ separated by 5 bp), which is perfectly conserved in rat, human, orangutan, dog and horse (Figure S5). It is therefore tempting to propose a regulation network for cloacal morphogenesis and gut development, in which *Hoxa13* and *Wnt5a* are instrumental upon regulation by ATRA-activated RARs.

### 4.3. ATRA Signalling in Vascular Remodelling

A role for ATRA-activated RAR in the development of embryonic vessels was inferred from the phenotypic analysis of mouse embryos deficient for ALDH1A2 or exposed to a pan-RAR antagonist [46,47]. However, it was widely believed, until now, that vascular abnormalities occurring in response to inhibition of ATRA signalling affect primarily pharyngeal arch arteries because of their unique situation, adjacent to neural crest cells and to the pharyngeal endoderm [11,12,47].

The present data reveal a key role of the RAR signalling pathway in the shift of the roots of the umbilical arteries from their original medial position to a lateral position. As documented from the study of vascular casts, this major event in remodelling the embryonic vasculature requires the coordinated outgrowth of capillaries, their fusion with others to form larger channels and the concomitant regression of the medial roots. It takes place in concert with an extensive remodelling of the arteries for the hindlimb buds that allows them to stay connected with the umbilical roots. It also allows the formation of the caudal branches of the aorta, i.e., the common iliac arteries from the lateral roots and the posterior mesenteric artery from the remnant of the medial roots [36,37]. In the absence of *Rars,* a single, large, median umbilical artery is formed. Such an abnormal vessel may, mechanically, impair the ascent of the kidney [36], and thus could be the cause of the pelvic kidneys always found in *Rarabg*^Δ*E10.5*^ mutants (Table 1). Moreover, the absence of formation of the common iliac arteries and of the inferior mesenteric artery could adversely affect the development of the structures that they normally supply, notably the cloaca and the urinary bladder (see below).

### 4.4. RAR-Dependent Vascular Defects as a Possible Cause of Anorectal Agenesis

Our data do not allow us to rule out a direct effect of the *Rarabg*^Δ*E10.5*^ mutation on the epithelium and mesenchyme of the cloaca, nor the possibility that RARs may be required independently for the morphogenesis of the cloaca and umbilical arteries. However, the consistent association of cloacal and arterial defects in *Rarabg^10.5^* mutants poses an interesting issue. Firstly, it is conceivable that the mesenchyme of the cloacal and umbilical regions may be part of a common developmental entity, because they are continuous with each other at the onset of cloaca development and appear to express a common set of genes, including *Rara* and *Rarb* [48], *Hoxa13* [49] and *Wnt5a* [50]. Along these lines, it is interesting to note that the belonging of the cloaca and the genital tubercle to the same morphogenetic field is often put forward to explain the systematic association of malformations of these two structures in other mouse models of ARMs [2,4,5,35].

Alternatively, all or part of the defects in the development of the cloaca of *Rarabg*^Δ*E10.5*^ mutants could be secondary to vascular defects. Along these lines, the absence of remodelling of the umbilical arteries in *Rarabg*^Δ*E10.5*^ mutants precedes the defects in cloaca development and causes the absence of (i) the arterial plexus, which derives from the posterior mesenteric artery and supplies blood to the developing rectum, and (ii) the iliac arteries, which supply the developing urinary bladder. Interestingly, absence of the posterior mesenteric artery was associated with congenital pouch colon, a form of human ARM [51,52,53]. Moreover, a recent large population-based study conducted in Norway found that pregnancies with a single umbilical artery have a strong association with anorectal atresia or stenosis [54].

The vascular hypothesis could account for the delayed outcome of the invalidation of the three *Rars* on cloacal development. Hypoplasia of the cloaca, anorectal agenesis and urinary bladder agenesis become respectively patent only 2, 3 and 4 days after invalidation of the *Rar*-coding genes. Moreover, it does not exclude the molecular actors proposed above (i.e., HOXA13, ALDH1A2 and WNT5A), which independently of one another have all been proposed to play roles in vasculogenesis or angiogenesis [46,55,56]. It is clear that further detailed studies on the relationships between control mechanisms for vascular remodelling and patterns of gene expression, cell proliferation and apoptosis in the cloacal region would be informative.

### 4.5. RAR Signalling and Human ARM

ARM are assumed to have a multifactorial aetiology, and a large variety of genetic and environmental factors have been implicated [1,7]. Fraser syndrome is one of the rarest causes [57]. Interestingly, the *Rarabg*^Δ*E10.5*^ phenotype strikingly resembles the Fraser syndrome, which consistently includes three major hallmarks (i.e., syndactyly, cryptophthalmos spectrum and urinary tract abnormalities), as well as three minor symptoms (i.e., anorectal defects, umbilical defects and nasal anomalies). This phenotype thus meets the current clinical diagnostic criteria proposed for this rare genetic disease [57]. The phenotypic similarities between mouse *Rar* mutants and human patients suggest that alterations in the ATRA signalling pathways might be at the origin of or might contribute to Fraser syndrome.

It was recently shown that the serum concentration of vitamin A in human ARM neonates was lower than that in control neonate [58]. These data and the fact that impaired RAR signalling pathways in rodents cause anorectal agenesis strongly suggest that maternal VAD, which remains a major public health issue in developing countries such as India, increases the risk of ARM in humans. In this context, it is worth noting that the occurrence of congenital pouch colon with no evidence for familial inheritance is much more important in India than in the rest of the world [51,52,53]. It is possible, therefore, that a dietary origin such as VAD may be responsible for the aetiology of this malformation.

### 4.6. Study Limitations

The 3D reconstruction approach that we used in this work consisted of colouring, by hand and individually, several dozen anatomical objects, on several dozen 2D HREM images, both in the plane of sectioning of the embryo and in its orthogonal planes. This non-automated approach is particularly time-consuming and, for this reason, is neither suitable for establishing normal (qualitative and quantitative) anatomical variations in a cohort nor for the systematic analysis of a large series of mutant embryos [59,60,61]. Our study shows instead that segmentation of HREM sections in early embryos is a valuable “secondary screen” for abnormal morphological phenotypes. Here, it allowed discovering a congenital anomaly which, so far, had gone unnoticed in mutants that we thought we knew perfectly well, as we have been analysing them for 25 years [11,27]. Our approach is complementary to conventional histology, which makes it possible to analyse cellular details and thus recognise defects of cellular arrangement within the tissues [11,12,27]. It is also complementary to other techniques of 3D morphological analysis, such as vascular corrosion casting or optical projection tomography (OPT), which, in the present study, would have been useful to visualise the capillary networks [37,62,63]. Even though “*where one part of a syndrome is never found except in the presence of another, and they are in the same anatomical region, a causal relationship may be suspected*” [36], the existence of causal connections between development defects in the cloaca and vascular system cannot be proved by a purely descriptive investigation. Molecular data are needed to confirm the scenarios proposed in the present article.

## Figures and Tables

**Table 1:** Abnormalities displayed by *Rarabg*^Δ*E10.5*^ mutants analysed at E14.5 on serial histological sections (n = 5, all females). # These abnormalities are completely penetrant. § These abnormalities equal or exceed, in frequency and eventually also in severity, those present in *Rara*^−/−^;*Rarb*^−/−^, *Rara*^−/−^;*Rarg*^−/−^ and *Rarb*^−/−^;*Rarg*^–/– KO^ mutants and are not observed in *Rarabg*^Δ*E11.5*^ mutants. §§ These abnormalities equal or exceed, in frequency and eventually also in severity, those present in *Rara*^−/−^;*Rarb*^−/−^ *Rara*^−/−^;*Rarg*^−/−^ and *Rarb*^−/−^;*Rar*^−/−^ KO mutants and are also observed in a milder form in *Rarabg*^Δ*E11.5*^ mutants. * This abnormality is completely penetrant and is as severe in *Rarabg*^Δ*E11.5*^ mutants.

Anorectal Agenesis #
**Urogenital defects**
• Agenesis of the urinary bladder # §
• Kidney hypoplasia # §
• Kidney ectopia # §
• Abnormal ureters # §
• Abnormal endings of the Wolffian ducts # §
• Agenesis of the Müllerian ducts # §
**Ocular defects**
• Cryptophthalmos # §§
• Short ventral retina # §§
• Ventral rotation of the lens # §§
• Coloboma of the optic disc # §
**Nasal defects**
• Shortening of the snout # §
• Choanal atresia # §
• Small nasal cavities # §
**Agenesis of salivary glands # §**
**Cardiovascular and respiratory defects**
• Myocardial hypoplasia #
• Persistent truncus arteriosus (1/5)
• Lung hypoplasia (1/5)
Syndactyly # *

## Data Availability

All data needed to evaluate the conclusions in the paper are present in the paper and/or the Supplementary Materials.

## Supplementary Materials

The following are available online at https://zenodo.org/record/4837423#.YNGd-OfgryQ, Figure S1: Left lateral views of the urogenital system and gut in control and *Rarabg*^Δ*E10.5*^ mutant littermates at E10.5, Figure S2: Left lateral and ventral views of the cloaca and arteries in control and *Rarabg*^Δ*E10.5*^ mutant embryos at E10.5, Figure S3: Left lateral and ventral views of the urogenital sinus, hindgut and arteries in control and *Rarabg*^Δ*E10.5*^ mutant embryos at E13.5, Figure S4: External appearance of control and a *Rarabg*^Δ*E9.5*^ mutant littermates at E13.5; Figure S5: RAR-binding sites at Hoxa13 and Wnt5a loci, Video S1: Control embryo at E11.5, Video S2: Control embryo at E12.5, Video S3: Control embryo at E13.5, Video S4: *Rarabg*^Δ*E10.5*^ mutant embryo at E11.5, Video S5: *Rarabg*^Δ*E10.5*^ mutant embryo at E12.5, Video S6: *Rarabg*^Δ*E10.5*^ mutant embryo at E13.5.
